# Assessment of bacteriological quality and safety of raw meat at slaughterhouse and butchers’ shop (retail outlets) in Assosa Town, Beneshangul Gumuz Regional State, Western Ethiopia

**DOI:** 10.1186/s12866-023-03106-2

**Published:** 2023-12-19

**Authors:** Mohammed Tesfaye Kebede, Asmamaw Abat Getu

**Affiliations:** Department of Biology, College of Natural & Computational Science, Assosa University, Assosa, Ethiopia

**Keywords:** Slaughterhouse, Butcher shop, Pathogens, Beef raw meat, Retail outlets, *Salmonella*, *Staphylococcus aureus*

## Abstract

Contaminated meat has been implicated in many cases of foodborne illness and poses serious challenges in developing countries. This study aimed to assess the quality and safety of raw beef meat in Assosa Town. The finding showed that the mean of Aerobic mesophilic bacteria (AMB) and *S. aureus* at retail outlets was 5.04 log10cfu/g and 3.84 log10cfu/g; 4.03 log10cfu/g and 3.5 log10cfu/g at slaughterhouse, respectively. The microbial load range of AMB at the butcher shop was 2.49-5.16 log10 cfu/g, while at the abattoir it was 2.75-7.52 log10 cfu/g out of 70 raw beef meat analyzed samples. Similar to this, the butcher shop and abattoir had *S. aureus* microbiological load ranges of 2.74 - 4.84 log10 cfu/g and 2.71-4.72 log10 cfu/g, respectively. In contrast, 25.7% and 34.3% of the samples in the abattoir and retail shop, respectively, were contaminated with *Salmonella sp*. For *S. aureus*, just 38.71% and 17.14%, respectively, of the samples at the retail and butcher shops were satisfactory. AMB found that 80% of the examined samples from butcher shops and 57.7% from abattoirs were satisfactory. Due to poor handling and environmental hygiene procedures by Assosa Town butchers, 77.1% of the meat contact surface and 82.9% of the carcass were exposed to flies. On the other hand, only 5.7%, 28.6%, and 22.9% of the butchers kept the carcass in the refrigerator, and wore gowns and hairnets, respectively. In slaughterhouses, the majority of respondents (87.5%) concur that there were certain challenges in achieving slaughtering in the working environment.

**Supplementary Information:**

The online version contains supplementary material available at 10.1186/s12866-023-03106-2.

## Introduction

Raw meat generally refers to any type of uncooked muscle tissue of an animal used for food and it supports the growth of both spoilage and pathogenic bacteria, this is because of its high moisture contents, a rich source of protein and fat, has fermentable carbohydrate, favorable pH and other growth factors [1]. According to the study conducted by Ahmad *et al*., [2], the *E. coli*, *S. aureus* and *Salmonella* were detected from total of 45%, 72% and 26% samples respectively. And 51% of beef meat samples had AMB more than 6 log10 CFU/cm^2^, which indicates highly contaminated meat and its possible role in spoilage and foodborne illnesses. On the other hands, study conducted on slaughtered beef meat quality in Jimma by Dabassa [3] revealed that the majority of beef meat samples had contaminant microorganism and some pathogens. In which aerobic mesophilic counts and *Staphylococci* varied from 0.19 to 3.67 log10 CFU/g and 0.95 to 2.28 log10 CFU/g respectively. Coliforms were present in all samples and *Salmonella species* in 10.4% of all the samples. Similarly, the prevalence of *Salmonella* positive in meat retail shop was 40.2% [4].

Potential biological hazards in meat include bacteria, toxins, viruses, protozoa, and parasites. Of the microbiological hazards, the most important are bacteria since bacteria cause a large proportion (approximately 90%) of all foodborne illnesses. Reduction of risk for human illness associated with consumption of raw meat can be better achieved through controlling points of potential contamination in the field, during harvesting, processing or distribution, or in retail markets, food service facilities, or in the home [5]. On the other hands, the microbiological quality of meat in retail shop depends mostly on the slaughter process, sanitation during processing, maintenance of adequate cold chain storage from the processing level and to the consumer and finally sanitation during handling at the retail end [6].

Moreover, the consumption of meat and meat products in Ethiopia has a very tidy association with cultural practices and religious beliefs, and are influenced by religions. Of all, cultural and religious considerations have always played a significant role in the preparation and consumption of meat products, and the stews are also made mainly from beef, lamb and chicken [7]. In turn, a large number of beef raw meat retail shops are available in Assosa Town and a great majority of consumers buy and eat beef raw meat in the form of traditionally named “*Ketefo, Kurt* or *Goredegored”* at which food hygiene and safety conditions are not assured and in which contaminated raw meat is one of the main sources of foodborne illness. Each stage in the lengthy chain between the time the meat is killed and when it is transported to retail outlets, where it is sold, may increase the danger of microbial contamination. Meat contamination by bacteria is also greatly influenced by the hygienic conditions at retail establishments and abattoirs in the area [8]. Regarding the safety and quality of raw meat in Assosa Town's slaughterhouse and retail stores, there is no information available. So that the current investigation was carried out to evaluate the safety and quality of the raw beef meat sold in Assosa town. The results of this study may provide the basis for the development of evidence-based treatments aimed at lowering hazards and improving food safety and hygiene protocols in slaughterhouses and retail stores. Lastly, the abundance of harmful microorganisms that should raise public health concerns in connection with raw beef meat in slaughterhouses and retail locations in Assosa Town is usefully documented by this study. It could also be used to raise consumer knowledge of the importance of food safety.

## Materials and methods

### Description of the study area

**Fig. 1:**
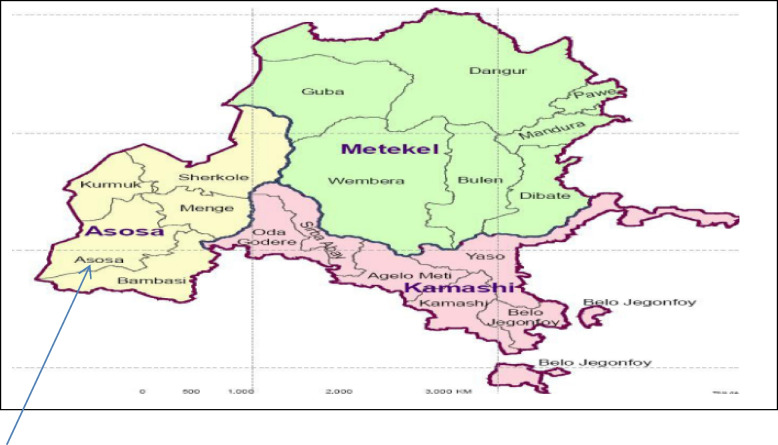
Map of the study area, Assosa Town in Benishangul Gumuz Regional State. Source: From Wikipedia, the free encyclopedia

The study was conducted in Assosa Town is about 661km far from Addis Ababa, the capital city of Ethiopia (Fig. 1). The study region located in north western part of the country between 09°17‟-12°06‟ north latitude and 34°10‟-37°4‟ east longitude [9] and having a total area of about 50,382 kilometer square [10]. The Benishangul-Gumuz region has a remarkable number of livestock populations with the current estimate of 659,587 cattle [11]. The bulk of the people mostly makes their living from mixed farming. One of the main advantages for the role of food supply is animal production. There are several endemic animal diseases caused by bacteria, viruses, protozoa, and parasites that compromise the productivity of livestock, and also common human diseases include diarrheal illnesses, tuberculosis, malaria, HIV/AIDS, and typhoid fever. There is one slaughterhouse in Assosa Town and has many partition and poor infrastructure and the health of the animal checked before slaughtering. The slaughtering started with the stunning of the animals by stabbing at the atlanto-occipital region using a sharp edge of knife, immediately followed by bleeding and removal of the head and the feet with the carcass in a horizontal position on the floor. The remaining slaughter steps (de-hiding, evisceration, post mortem inspection and carcass labeling) were performed in vertical position after manually hanging the carcass by hooks and sliding it over the rail system. Finally, the carcasses were carried by the slaughters and laydown the car transported to retail outlets.

### Study design and period

A cross-sectional and experimental study was conducted from May 2018 to February 2019 to assess the bacteriological quality and safety of raw beef meat at slaughterhouse and retail shops as well as to assess the handling practices of raw beef meat in Assosa Town.

### Sampling size and sample collection

A total 70 raw beef meat samples in which 35 each were collected from different butchers shop and slaughterhouse using sterile glass containers and all the samples was transported to Assosa University Biology laboratory and stored in refrigerator until microbiological analysis was done. The study retail shops should be selected randomly.

### Sample preparation

Microbiological analysis was carried out as described by the methods of Fowel and Oso [12]. Twenty five grams (25g) of the raw meat sample was chopped and mixed with 225 ml of sterilize buffered peptone water (Oxoid LTD., England) for 5 min in sterilized flask followed by ten-fold serial dilutions (10^-1^ to 10^-4^) of homogenates were prepared and subjected to the enumeration of AMB, *S. aureus,* total coliforms and isolation of *E.coli* and *Salmonella spp*.

### Bacteriological analyses of raw beef meat sample

**Enumeration of Aerobic Mesophilic Bacteria (AMB)**

Aerobic mesophilic bacteria (Aerobic mesophilic count) were carried out on plate count agar as described by APHA [13]. Samples were serially diluted and an aliquot of 1 ml of each serial dilution was transferred to the pre dried duplicate petri dishes and plate count agar (15-20 ml) was poured on each plates. Plates were gently swirled to uniformly mix the sample and incubated at 37ºC for maximum of 48 hours. After incubation AMB was determined from appropriate plates and result was reported as log cfu/g [13].

**Enumeration of** ***Staphylococcus aureus***

Enumeration of *S. aureus* was done by spreading an appropriate dilution of sample on mannitol salt agar plates followed by incubation at 37°C for a maximum of 48 hours [13]. Yellow or orange colonies surrounded by yellow zones due to mannitol fermentation was enumerated and reported as mean log cfu/g of food.

**Total coliforms count**

Test for the presence of total coliforms based on the procedure described in the Manual of Food Quality Control of FAO [14]. From serial dilution (10^-1^, 10^-2^ & 10^-3^), one milliliter of each dilution was inoculated into triplicate (3 test tubes) tubes containing sterile Lauryl Tryptose Broth (Blulux Laboratories (p) Ltd, India) with inverted Durham tubes and incubated at 37°C for a maximum of 48 hours. Then for confirmatory test, gas positive lauryl tryptose broth tubes at the end of the incubation period was gently agitated and loopful of each culture was transferred to tubes of brilliant green bile (2%) broth (Oxoid, England) with inverted Durham tubes and incubated at 37°C for a maximum of 48 hours. Tubes which formed a gas as well as color change as a result of total coliforms was reported based on standard statistical tables as the most probable number (MPN) per gram of meat sample.

**Isolation and identification of** ***E. coli***

For the isolation of *E. coli* 1 ml of each serial diluted sample was transferred into duplicate sterile Petri dish which contain Mac Conkey agar medium and incubated at 37˚C for 24 hours. For purification and refreshment purposes, suspected typical colonies having bright and pink color was streaked on a nutrient slant and incubated for additional 24 hours. Then, transferred to Eosin methylene blue agar (EMB) and *E. coli* was confirmed by its transparent green metallic sheen color. A loopful (representative colony) from culture on EMB agar was inoculated tube containing Tryptone water and incubated at 44°C for 24 hours. The formation of indole detected by the addition of Kovacs reagent (approximately 0.1 ml and mix gently) to tryptone water then the presence of indole is indicated by a red color in the Kovacs reagent, forming a film over the aqueous phase of the medium. Confirmatory tests positive for indole, metallic sheen on EMB agar show the presence of *E. coli* [13].

**Isolation of** ***Salmonella***

Twenty five gram of each beef meat sample was blended and homogenized in 225 ml buffered peptone water (BPW) and incubated 24 hours at 37°C as pre-enrichment for *Salmonella*. Pre-enrichments, which entail inoculating 10 milliliters of the Rappaport visiladis broth (RVB) with 0.1 milliliter of the pre-enriched sample, are used in conjunction with selective media enrichment to increase *Salmonella* recovery. Samples on SC broth and RVB broth were incubated at 37°C and 42°C for 24 hours, respectively. Enriched *Salmonella* cultures were streaked onto Xylose Lysine Desoxycholate (XLD) agar and Brilliant green bile broth (BGB) and incubated at 37°C for 24 hours. Typical colonies grown on XLD agar having transparent zone of reddish color (red colonies) with or without a black center and colorless or white colonies on BGB due to the color change of the media were suspected for *Salmonella* [14].

#### **Biochemical test for *Salmonella***

After incubation on nutrient agar different biochemical tests on Triple Sugar Iron (TSI) slant, Voges-Proskauer (Vi) broth, Lysine Iron (LI) agar, Indole (I) broth, Methyl Red (M), Citrate (C) utilization were done according to Mooijman *et al.,* [15] and incubated at 37 °C for 18-24 hours and checked for confirmation [16].

**Assessments of knowledge, hygienic and handling practices of workers at different retail outlets and slaughterhouse**

The observation checklist and semi structured questioner as well as interview was used for assessing of the sanitary conditions and meat hygienic practices of butchers and workers in slaughterhouse. A check lists covering topics on the personal hygiene and practices of the butchers and hygiene of the butchers shop and slaughterhouse to assess whether the area exposed to flies, insects and animals, presence of solid and liquid waste. Total of 41 respondents in which 35 and 16 participated from retail outlets and slaughterhouse, respectively.

**Ethics approval**

The study was carried out after obtaining ethical clearance from Health Science College of ethical approval committee of the Assosa University.

**Data analysis and interpretation**

Mean bacterial number were compared by one way ANOVA and independent samples t test using SPSS software 20 to determine if microbial significant differences in raw beef meat samples among the various retail shops and abattoir (slaughterhouse). Significance of differences was held at *p* value less than 0.05. Descriptive statistics also include frequency distribution and percentages.

## Results and discussion

### Enumeration and satisfactory level of Aerobic Mesophilic Bacteria (AMB)

Plate count of aerobic mesophilic microorganisms found in meat is one of the microbiological indicators for food quality. The presence of aerobic organisms reflects existence of favorable conditions for the multiplication of microorganisms. In the present study revealed that among the total of 35 raw beef meat samples in retail outlets; 26(74.3%) were counted as contaminated by AMB with minimum and maximum value of 2.75 and 7.52 log cfu/g, respectively (Table 1), whereas among samples analyzed at slaughterhouse; only 20(57.1%) were contaminated and counted as AMB with minimum and maximum value of 2.49 and 5.16 log cfu/g, respectively. The mean ± SD of viable bacteria (AMB) isolates from abattoir and butchers shop was 4.03 log10cfug±0.90 and 5.04 log10cfu/g ±1.41 respectively (Table 1). As the *p* value showed that (Table 1) there was significance differences of AMB counts between retail outlets and abattoir, i.e., *p* =0.008.

**Table 1:** Mean loads (log10 cfu/gm) of aerobic mesophillic bacteria in raw beef meats collected from slaughterhouse and retail outlets, Assosa Town, 2018

**Raw beef meat site**	**No. of total samples**	**No. of positive samples**	**%**	**Minimum**	**Maximum**	**Mean**
S. house	35	20	57.1	2.49	5.16	4.03
R. outlets	35	26	74.3	2.75	7.52	5.04
**Total**	**70**	**46**	**65.7**	**2.49**	**7.52**	**4.60**

S. house* Slaughterhouse, R. outlets*Retail outlets, SD*Standard Deviation

**Fig. 2:**
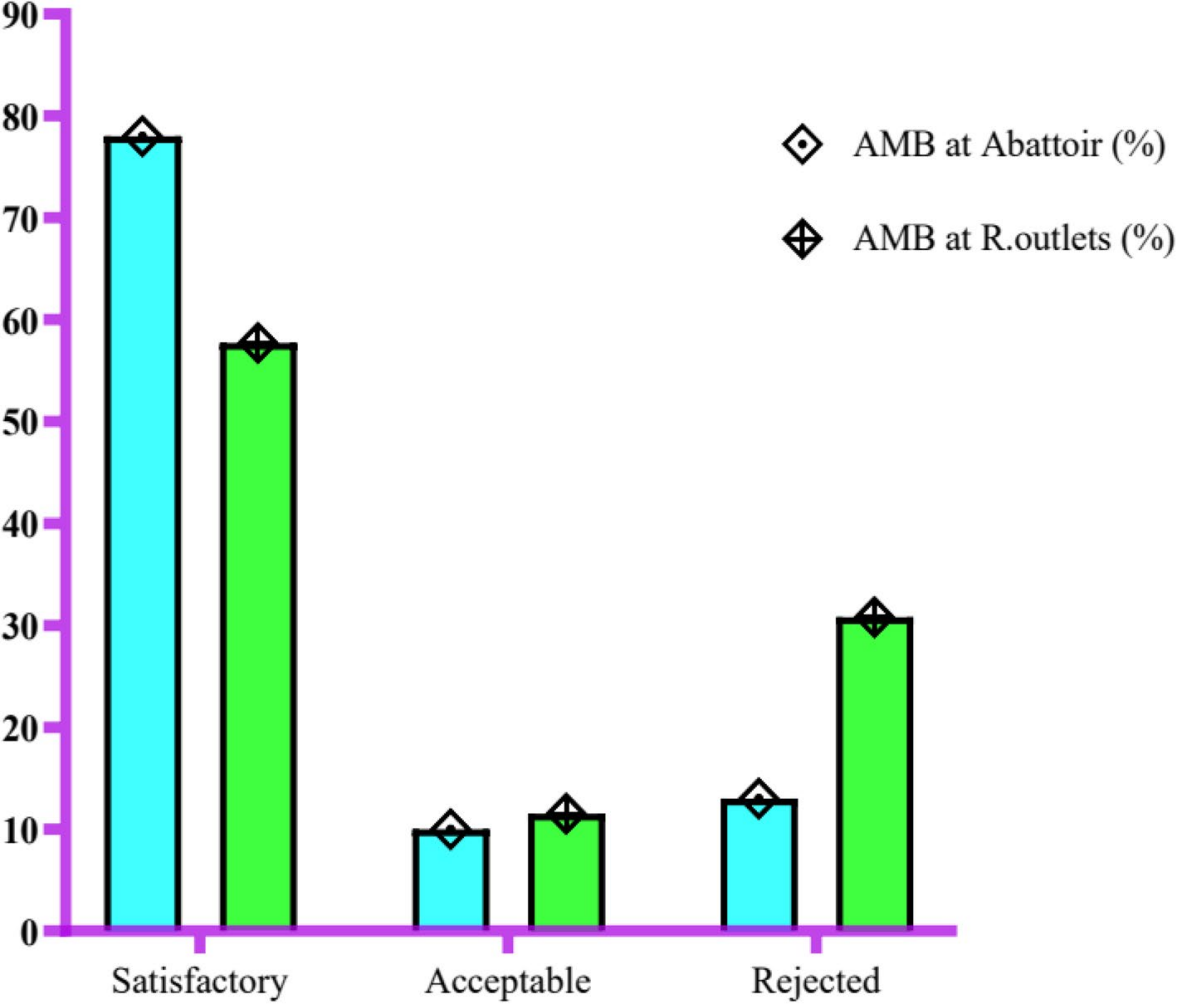
Aerobic mesophilic load in abattoir and retail outlets, Assosa Town, 2018 (*n* = 70)

On the other hands, microbiological quality of AMB counted at abattoir and retail outlets showed that 78% and 57.7% of the samples were satisfactory level, respectively (Fig. 2) because of microbiological quality ranged less than 1×10^5^cfu log/g, which indicates good microbiological quality. Similarly, 2 (10%) and 3 (11.5%) samples taken from abattoir and retail outlets was counted as marginal acceptable, respectively, and the remaining 13% and 30.8% out of each of 35 raw meat samples at abattoir and retail outlets were reported as rejected, respectively (Fig. 2) in which their microbiological level ranged less than 1×10^6^ and equal or greater than 1×10^6^ cfu log/g categorized as marginal acceptable and rejected, respectively [17, 18].

Large aerobic colony counts (5 log cfu g-1) make food dangerous and suggest improper handling, storage, or general cleanliness, yet samples have inferior microbiological quality as a result. Even though the AMB of any food items is not a guarantee of their suitability for eating, it is crucial for assessing the hygienic conditions in which food has been grown, handled, and kept [19]. As a result of the food's generally unclean quality and high occurrences of bacterial contamination [18], these issues are mostly to blame. High AMB could be a sign that the temperature and length of time controls in storage or exhibition facilities weren't sufficient to stop bacterial development [20].

As compared the current study of mean AMB value obtained in retail outlets with results of other studies, the present study was higher than that was reported by Cho *et al.,* [21] for raw meats in Korea with the mean ± SD value of 4.71±1.53 log cfu g-1. A similar study was carried out in Lagos [22]; the total aerobic bacteria count ranged from 3.3x10^3^ to 5.9x10^6^ CFUg-1. On the other hands, the study conducted in Nigerian butchers shop reported by Ologhobo *et al.,* [23], the highest APC was 6 log cfu/g. Moreover, waste water and garbage discarded in the streets and foods such as meat are not effectively protected from dust and flies. It was observed that raw meats were left uncovered and exposed to microbial contaminants during the entire selling period in the butchers shop. These factors are likely to be linked to the high aerobic plate figures recorded in present study.

### Enumeration and satisfactory level of *Staphylococcus aureus*

*Staphylococcus aurous* is the most prevalent and economically significant pathogen cause infections in meat ruminants. It is a pathogenic bacterium that induces several of human illnesses. The ability to cause a wide range of diseases may be associated with its production of a large spectrum of extracellular toxic compounds and other virulence factors such as toxic shock syndrome, exotoxins and enterotoxins [24].

The present finding showed that, 29(82.8%) and 19(54.3%) of raw meat samples were contaminated and counted to *S. aurous* at butchers shop and abattoir with mean value of 3.84 and 3.50 log_10_cfu/g, respectively (Table 2). However, the mean values of the present samples were by far greater than that reported for meat obtained at retail shop, i.e., 2 log cfu/g [25]. Khalafalla *et al*., [26] also reported lower counts of *Staphylococci*, i.e., 3 log cfu/g in ground beef meat samples.

**Table 2:** Mean load (log10 cfu/gm) of *Staphylococcus aureus* raw beef meat collected from slaughterhouse and retail outlets, Assosa Town, 2018

**Raw meat site**	**No. of carcass tested**	**No. of positive samples**	**%**	**Minimum**	**Maximum**	**Mean± SD**	***P* value**
S. house	35	19	54.3	2.71	4.72	3.50 ± 0.54	
R. outlets	35	29	82.8	2.74	4.84	3.84 ± 0.61	**0.170**
**Total**	**70**	**48**	**68.5**	**2.71**	**4.84**	**3.70 ± 0.60**	

S. house*Slaughterhouse, R. outlets*Retail outlets, SD*Standard Deviation

The minimum and maximum load of *S. aureus* at retail outlet was 2.74log_10_cfu/g and 4.84log_10_cfu/g, respectively, and similarly, the minimum and maximum load of *S. aureus* at abattoir load was 2.71log_10_cfu/g and 4.72log_10_cfu/g, respectively (Table 2). While, as the table showed that (Table 2), there is no statistical differences of mean microbial load of raw meat at abattoir and butchers shops (*p*=0.170).

**Fig. 3:**
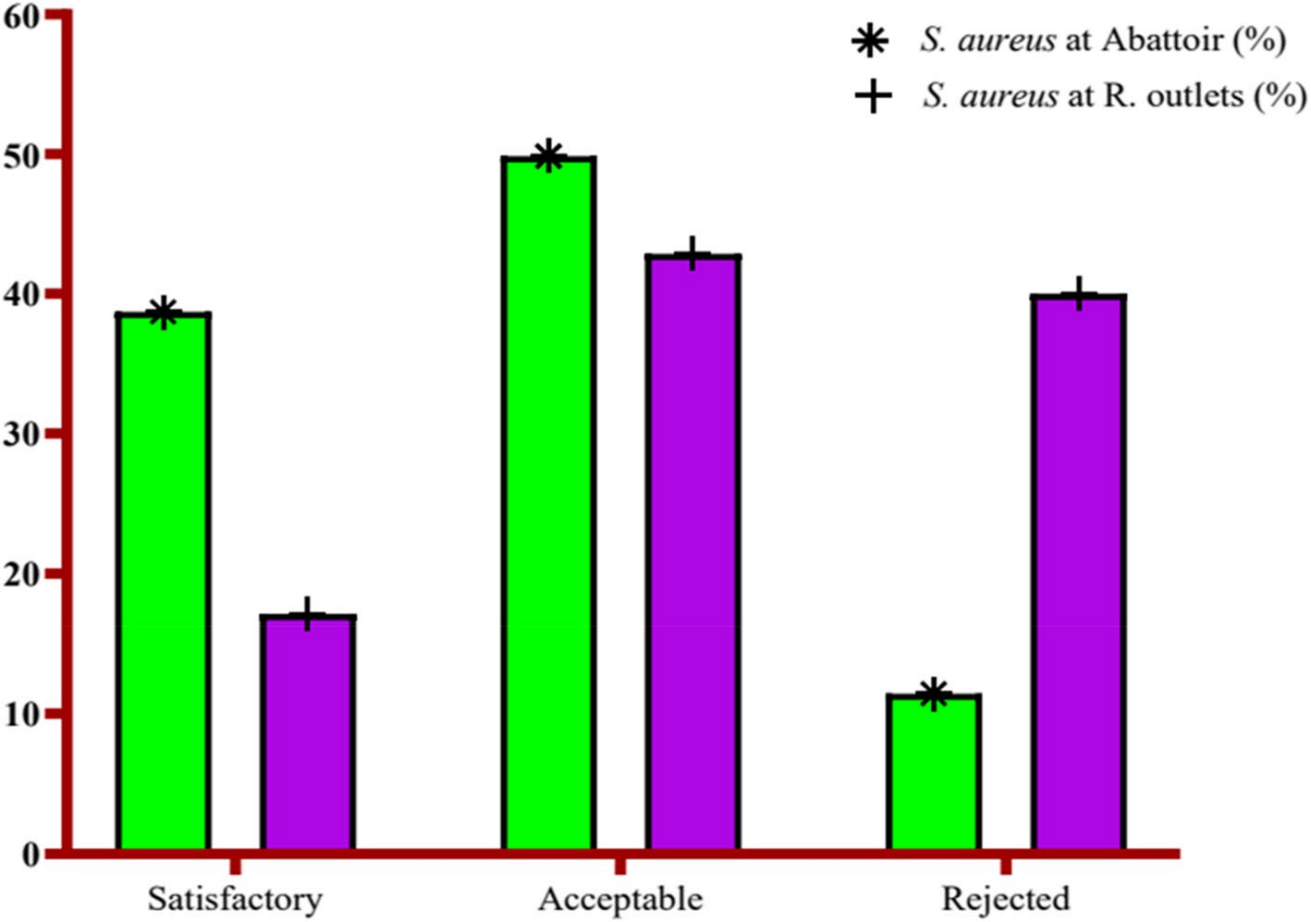
The load of *S. aureus* in abattoir and retail outlets, Assosa Town, 2018 (*n* = 70)

As it is shown (Fig. 3), 4(11.43%) and 14(40%) of the raw beef meat samples from abattoir and butchers shop were unsatisfactory (rejected) levels, respectively, results were outside of acceptable microbiological limits (ranged ≥10^4^cfu/g) and potentially hazardous for consumers. The microbiological quality ranged ≥10^4^cfu/g due to inadequate temperature control and poor hygienic practices. The levels in this range may cause food borne illness and immediate remedial action should be initiated. 18 (49.86%) and 15 (42.86%) samples at abattoir and retail outlets, respectively, were reported as marginal level (Fig. 3), which their microbiological quality ranged between 10^2^-10^3^cfu/g results are borderline in that they are within limits of acceptable microbiological quality but may indicate possible hygiene problems in the preparation of the food. However, 13(38.71%) and 6(17.14%) of raw meat at abattoir and retail outlets respectively were with satisfactory level (Fig. 3) which range less than 1×10^2^cfu/g.

As compared the present finding to the results of other studies, unsatisfactory levels of *S. aureus* in retail outlets (40%) were higher rate in this study. For instance, in a study, from 200 samples of street vended ready-to-eat meats sold in Cameroon, 20(10%) were contaminated with *S. aureus* [27]. Similar other study conducted in Taiwan, *S. aureus* were detected with unsatisfactory levels of 17 % of the total sample [28].

The high number of *Staphylococci* particularity in retail outlets in the present study indicates that *S. aurous* is a typical bacteria found in unprocessed meat that is handled with bare hands. Contamination with *S. aurous* may result from the origin of the meat or from poor hygiene conditions, and through the hands or skins of handlers (human beings), hand touch because of improper handling activities, as they are typical contaminants from hands, discharge from human, and clothing, utensils, and the temperature time abuse before consumption could lead to further proliferation of the bacteria [29]. Furthermore, the majority of butchers lacked aprons, masks, and gloves. Food handlers' hands are their most essential body part and the primary source of cross contamination. Occasionally, food handlers are unaware of their own actions and may rub their faces, noses, and other body regions. Adults contain *S. aureus* in their respiratory passages, skin, and superficial wounds due to the presence of cross contamination, which is typically associated to human skin. As a result, droplet infections that are present in coughs and sneezes may easily spread to both the environment and the food being touched [30].

### Enumeration of total coliforms, isolation and identification of *E.coli* and *Salmonella sp.*

**Fig. 4:**
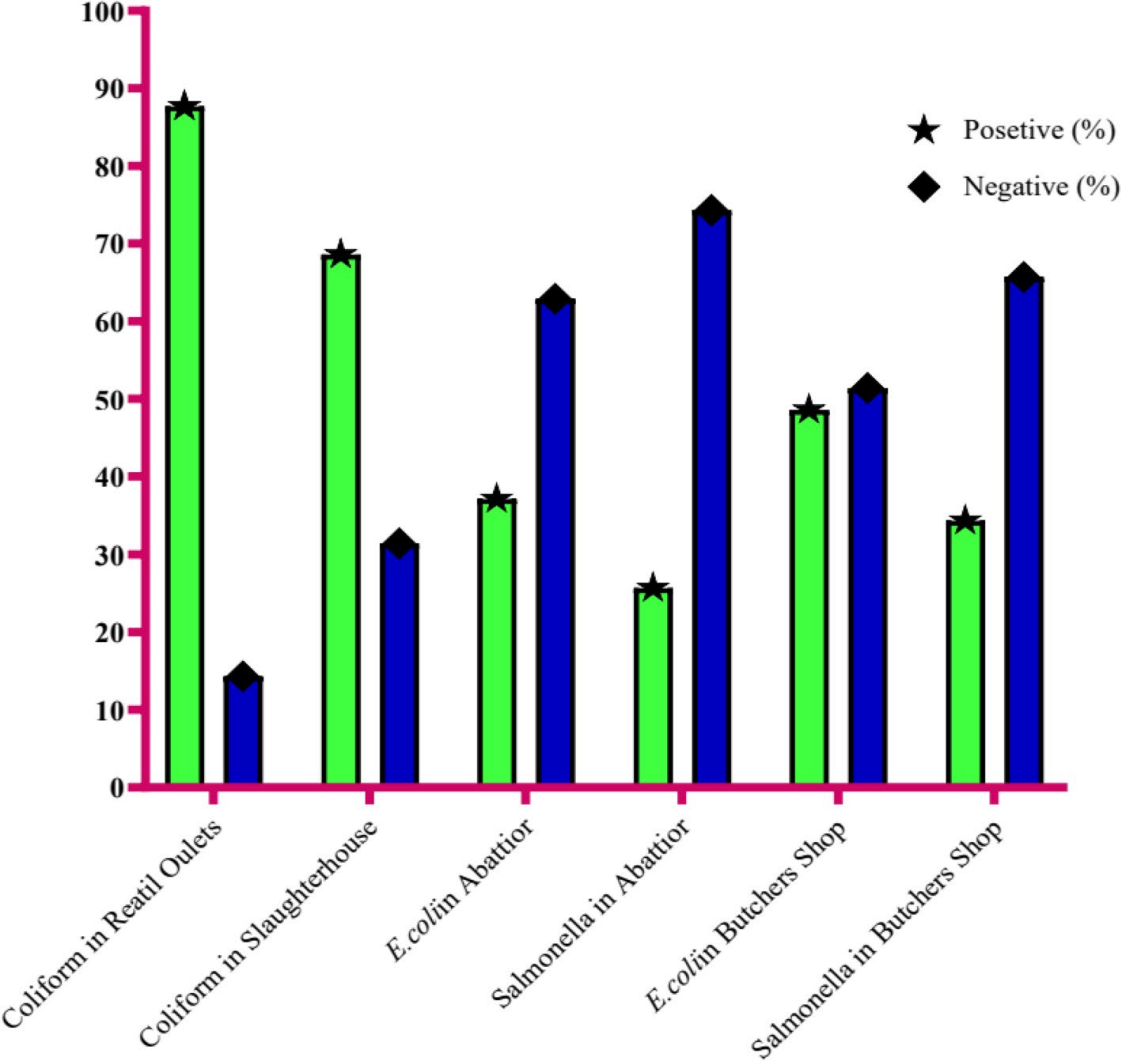
Total coliform, *E. coli and Salmonella* contamination of the raw beef meat at abattoirs and retail outlets

Among the total of 70 samples, 35 each of raw beef meat collected in different sites of retails and abattoir, 17(48.6%) and 13(37.1%) were respectively contaminated with *E.coli* (Fig. 4). The presence of *E.coli* in raw beef meat at point of sale can significantly contribute that poor food handling practices and furthermore its detection indicates a recent faecal contamination through poor sanitation practices of food vendors and also indicates the possibility of contaminating enteric pathogens [31, 32].

In addition, *E. coli* is, furthermore, a known causative agent of diarrhea and other foodborne related illnesses through the ingestion of contaminated foodstuffs. Pathogenic members of the *E. coli* form group as well as the entero-bacteriacae family are represented by genera such as *Salmonella* and *Shigella* and are found in the intestines of humans and animals [33]. On the other hands, when the current study was compared with other studies, nearly incidence of *E. coli* was found at retail outlets, for instance, according to the study conducted by Giri *et al*., [34] reported that 16(29.09%) samples out of 55 samples were contaminated with *E. coli;* similarly the present result was higher than those obtained in Korean from street vended raw meats, whereby 9(45%) of street-vended raw meat had contaminated with *E. coli* [21]. Another study in Mexico conducted by Diaz-Lopez *et al.,* [35] showed that *E. coli* was detected in 37 of 43(86%) from street vended meat, and this was so higher *E.coli* contamination level than the present study at both abattoir and butcher shops.

In general, *E. coli* is one of the bacteria that are part of the healthy intestinal micro-flora of people and warm-blooded animals. The way these meats are produced, handled, sold, and transported is totally dependent on tradition. As seen during sample collection, handling meat with bare hands, not wearing an apron, not covering one's hair, and handling money while serving may also be contributing factors to unsanitary circumstances. Such a system might provide a favorable environment for the infection of *E. coli* and other pathogens [36, 37].

Similarly, as shown as Fig. 4, the percentage of positive samples of *Salmonella* contamination were; 9(25.7%) and 12(34.3%) isolated at abattoir and retail outlets, respectively. In line with CDC (Center for Disease control and prevention), *Salmonella* is one of the most common case of serious foodborne illnesses and certain strains are of significant importance due to the emerging resistance to common antibiotics [8]. Salmonellosis often found in raw meat due to contamination caused through poor personnel hygiene and the use of contaminated equipment’s. Cutting board surfaces used for preparation of meat and equipment like meat grinds, mincers blenders are considered an important source of meat contamination by *Salmonella*. Its presence in meat can be explained by inadequate evisceration or slaughter of sick animals. The presence of *Salmonella* in the ready to eat meat represents a great hazard for the consumer because those pathogens are often responsible for gastroenteritis, food poisoning, typhoid, and paratyphoid fever [38].

It was found that prevalence of *Salmonella sp.* contamination at abattoir in the present study was 9(25.7%) out of 35 raw beef meat samples. However, the presence of even small numbers of *Salmonella* in carcass meat may lead to heavy contamination of meat. When the present study was compared with other studies, it had higher prevalence of *Salmonella*, for instance, as compared to other findings conducted in Jimma town, 2(1.2%) samples was contaminated with *Salmonella* and 5(8.3%) quite far as compared Tasew *et al*., [39]. Whereas the rate of Salmonella isolation from raw meat at retail was 20% in Gaborone, Botswana [40] 9% in raw meat obtained from butchers shop in Awassa [41] and 42% in Addis Ababa [42]. Also a study was conducted in Taiwan, 41% of the raw meats were contaminated with *Salmonella sp*. [28] which is so exceeds than the result of the current study.

*Salmonella sp*. contamination of raw meat is frequently related to inadequate refrigeration, subpar sanitation, and subpar personal hygiene. Therefore, handling meat by individuals who are *Salmonella* carriers may contribute to the proliferation of this bacterium in meat. The presence of the organism is alerted by food handlers' inappropriate handling of the meat and utensils during preparation and serving [43] . This is in line with the findings of Molla and Mesfin [44], which suggested that the spread of *Salmonella* to uncontaminated carcasses could also occur through the cross-contamination of worker hands, tools, and utensils. On the other hand Mohammed [45], emphasized that *Salmonella* constitutes a concern to humans, and public practitioners should take into account potential mechanisms of Salmonella transmission in meat during slaughtering and preparation is more common [24].

To identify the isolated bacteria from raw meat at slaughter house & retail outlets by morphological and biochemical characteristics are necessary. Using different parameters identify the isolated bacteria as *Salmonella, S. aureus and E.coli* see the detail in Table 3.

**Table 3:** Morphological and biochemical characteristics of bacteria isolate from raw beef meat from slaughterhouse & retail outlets

**Parameters**	**Isolated microorganisms**		
	***Salmonella***	***S. aureus***	***E.coli***
Growth in Mannitol salt agar	N/A	Bright yellow (orange)	N/A
Growth in MacConkey agar	-	N/A	Red/pink
Grams reaction	-	***+***	**-**
Cellular morphology	Rod (Flagellated)	Cocci	Straight Rod
Coagulase test	-	***+***	**-**
Growth on TSI (Triple sugar iron agar)	Butt – Black	N/A	Slant – Red, Butt –Yellow
Growth on Lysine iron agar(LIA)	Butt – Yellow	N/A	Butt & Slant – Red
Sugar fermentation	-	+	+
H_2_S production	+	N/A	-
Gas formation	+	N/A	- (+)
IMViC test			
Indole test	-	-	+
Methyl red	+	N/A	+
Voges-proskauer	-	N/A	-
Citrate test	+	-	-

+* Positive (grown), -*Negative (not grown), N/A* not applicable

### Assessments of butchers’ knowledge in relation to foodborne diseases in Assosa Town (*n*=35)

In the present study the educational status or level of the respondents of meat vendors 10(28.6%), 12(34.3%), 5(14.3%) and 8(22.86%) were illiterate, elementary, high school level, and college respectively (Table 4), this showed that most vendors are relatively educated. On the other hands, in regard to knowing about foodborne disease; 21(60%) respondents had consider knowledgeable to foodborne disease and the remaining 14(40%) were not; this situation may enhance the risk factor to contaminate the street vended meat in present study. This result is contradicted to the findings of Ehiri *et al., *[46] indicated in their study that most of the vendors 56(70%) respondents who took part in food hygiene education in Scotland and knew about the foodborne disease.

**Table 4:** Knowledge of butchers’ in relation to foodborne diseases in Assosa Town, 2018 (*n*=35)

**Parameters**	**Frequency *n*=35**	**Percent**
Educational status		
Illiterate	10	28.6
Elementary	12	34.3
High school	5	14.3
College/University	8	22.9
Do you know about food borne disease?		
Yes	21	60
No	14	40
Have you taken any training on food hygiene and safety?		
Yes	14	40
No	21	60
Do you work when you have diarrhea?		
Yes	18	51.4
No	17	48.6
Do you know reason for food contamination?		
Yes If yes, please specify……	26	74.3
No	9	25.7
Do you know that food borne diseases are preventable?		
Yes (If yes, how ……)	29	82.9
No	6	17.1
Do you agree that raw meat can be contaminated through cross contamination with Handlers?		
Strongly agree	9	25.7
Agree	12	34.3
No opinion	2	5.7
Strongly disagree	12	34.3
Food borne pathogens can be seen by naked eyes?		
Yes	17	48.6
No	18	51.4
Are insects such as cockroaches and flies might transmit food borne pathogens?		
Yes	26	74.3
No	9	25.7
Apparently healthy food handlers might carry food borne pathogens?		
Yes	11	31.4
No	24	68.6

Lacking in personnel hygiene among food handlers is one of the most commonly reported practices contributing to food borne illnesses [47]. Training is crucial prerequisite to successful implementation of a food safety management system and food safety practices will only be implemented given adequate resources and appropriate management culture [48]. However, in the present study 21(60%) respondents were not taken any training but the remaining 14(40%) of them could take training. Additionally, 18(51.4%) respondents agreed that vendors should be prevented from vending if they were sick by diarrhea and the remaining 17(48.6%) does not agreed (Table 4).

Meat is considered to be spoiled when it is unsuitable for human consumption and based on the present study 26(74.3%) respondents had knowledge about a given meat might be contaminated with vectors or flies, for example, when the vendors were asked or interviewed in the study site, they were specify some reasons in which such contamination can be caused by a wide variety of factors or reasons, such as improper handling and practices, exposure to open air or by flies and cockroaches which carry the most common pathogenic microorganisms. On the other hands, the 9 out of 35 were non knowledgeable with idea how meat could be contaminated and unfortunately 21(60%) respondents were disagree with idea that infected carrier butcher cause foodborne illness (Table 4).

The majority of the respondents; 29(82.9%) know foodborne disease are preventable and they give the following reasons; most meat-borne outbreaks are most serious difficult but it is easy to prevent the spread of many types of infection for instance hand washing. This is because the hands of food handlers can be as vector to spread harmful microorganism through cross contamination (Table 4). To sum up the present study, street vended meat in the study site were displayed and sold openly at very dirty surrounding on the road side. This can easily be contaminated by dust, insects, such as cockroaches and flies and those might transmit and enhance the level of food borne pathogens and those 26(74.3%) respondents (butchers) did agree with this statement and the remaining 9(25.7%) of them could not. In addition to this 11(31.4%) respondents had good knowledge about healthy food handlers might carry food borne pathogens and unfortunately 24(68.6%) had not knowledge (Table 4).

Observational study was also used in the assessment of food safety practices by street vendors during their trade and the environment or vending site assessment. In which the observation showed that the raw meats were displayed uncovered for more than six hours for sell at ambient temperature on a table or a carton which would be used again and again, and majority vendors was located very close to the main road. The hands of food handlers are an important vehicle of food cross contamination. In which 29(82.9%) meat retail outlets were exposed to dust and harbor vector such as flies and again majority of food vendors in the street had direct physical contaminants with the raw meat. On the other way, vendors could handle money when vending meat and this practice leads to contamination of raw beef meat from dirty money through cross contamination. Similarly study conducted by Temeche *et al.,* [49] found that 35% vendors due to the bare hand contact with meat as a contributing factor with handling money. The sanitary condition of the vending environments also was poor as observed in the current study site (Table 5).

**Table 5:** Assessments of beef meat vendor’s handling practices and surrounding environments of retail outlets, Assosa Town, 2018 (*n*=35)

**Characteristics**	**Frequency**	**Percent**
Cleaning status of meat contact surface, equipment or tables		
Protected well	8	22.9
Unprotected	27	77.1
Food handlers (butchers) in retail shops wear gowns appropriately?		
Yes	10	28.6
No	25	71.4
Food handlers (butchers) in retail shops wear a hairnets?		
Yes	8	22.9
No	27	77.1
Finger nails of the meat handlers?		
Clean & trimmed	9	25.7
Not trimmed & unclean	27	74.3
The carcass is stored and kept properly in refrigerator?		
Yes	2	5.7
No	33	94.3
If any contact of the carcass with the bare hands of the butchers?		
Yes	35	100
No	-	-
Is their proper solid /liquid/ waste storage receptacle near the vending site?		
Not available	30	85.7
Available (proper)	3	8.6
Available but improperly	2	5.7
Is the vending area with cleaned floor, wall and adequate lighting?		
Yes	15	42.9
No	20	57.1
Is the carcass in retail shops (outlets) easily exposed to harbor vectors such as flies?		
Yes	29	82.9
No	6	17.1
Is there any discharging from vender nose, eye, ear or cough during visit		
Observed	3	8.6
No Observed	32	91.4
Vendors handling money when vending the raw meat?		
Yes	29	82.9
No	6	17.1
What look like the general hygiene situation of retail shop?		
Clean (Satisfactory)	11	31.4
Not clean (Un satisfactory)	24	68.6

All food handlers have a basic task to maintain a high degree of personal cleanliness and observed hygienic and safe food handling practices. Keeping hands clean, shortening fingernails, wearing clean working garment and hair cover (hair net and cap) are some of the precautions that a food handler must maintain [50]. The hands are the most important vehicles for the transfer of organisms from faeces, nose, skin, or other sites to food. Because the organisms such as *Salmonella typhi*, non-* Salomnella typhi*, *Compylobacter spp*. *E. coli, S. aureus* and other microbial spp*.* can survive on fingers tips and other parts of the body [51].

Wearing clean working garment and hair cover (hair net and cap) are some of the precautions that a food vendors must maintain [50]. In the present study no one of the vendors in retail shops had access to wear hand glove and only 8(22.9%) wore of hairnets (Table 5). When the present study was compared with other study conducted by Çakiroglu and Ucar [52] found that 82.9% of the staff wore caps, masks and gloves during food production. Because hair is known to harbor *S. aureus,* so it is essential to prevent loose hair and dandruff from falling onto the food or food preparation areas by having head or hand cover. Furthermore as shown as (Table 5), 20(57.1%) of street meat vendors they achieved their activities under inadequate lighting, clean floor and wall. In most cases 33(94.3%) meat carcasses were exposed to room temperature because of there had no refrigeration/cooling facility to stored and kept it. As a result, due to lack of refrigeration the marketing process was open and the meat could be contaminated through different pathogens. And also assessment of the cleaning status of meat surface, equipment or tables revealed that 27(77.1%) of them being regarded as poor or unprotected well and only the remaining 8(22.9%) as good or protected well (Table 5).

To sum up, all food handlers should have a basic task to maintain a high degree of personal and environmental cleanliness of the retail establishments, however, in regard to this only 11 (31.4%) of retail shops looks like clean, and as observation showed that, the majority 24(68.6%) of the hygienic status of retail (butchers) shops were look like not clean or unsatisfactory.

### Hygienic practice and knowledge of slaughtering workers and the surrounding environment of the slaughterhouse in Assosa Town, 2018

The majority of the slaughterhouse workers were educated, as seen by Table 6 above, with 62.5% having completed elementary school, 31% having completed high school, and 6.25% being illiterate. Only 2(12.5%) of the slaughtering workers had taken training on food hygiene and safety and what makes the problem as serious about 56.5% of workers in the slaughterhouse did not check their health status recently. Even near to half of the respondents (43.75%) in the slaughterhouse they did not know about the reason for food contamination. However, most of the slaughtering workers had well understanding about if consumption of raw meat may leads to food borne disease and again they agreed that most of food borne is preventable. 3(18.75%) and 2(12.5%) of the respondents were said as “strongly disagree” and “they did not have any opinion”, respectively concerning if meat can easily cross contaminated through food handlers (Table 6). Similarly, most of slaughtering workers knew (agreed) that insects or flies can easily transmit food borne pathogens. In the above (Table 6) as it was observed, majority (81%) and 25% of slaughter working did not wear hand glove and hairnet (gown), respectively. Additionally, a tanker served as the source of water utilized in the slaughterhouse to wash the carcasses (water container).

**Table 6:** Slaughtering workers knowledge and handling practices in Assosa Town, 2018

**Characteristics**	**Frequency *n*=16**	**Percent**
Educational status		
Illiterate	1	6.25
Elementary	10	62.5
High school	5	31.25
Preparatory	-	-
College	-	-
Do you know about food borne disease?		
Yes	11	68.75
No	5	31.25
Have you taken any training on food hygiene and safety?		
Yes	2	12.5
No	14	87.5
Do you work when you have diarrhea?		
Yes	1	6.25
No	15	93.75
Are you examined your health status recently?		
Yes	7	43.75
No	9	56.25
Do you know reason for food contamination?		
Yes	9	56.25
No	7	43.75
Do you believe that food borne disease caused by consumption of meat (raw meat)		
Yes	13	81.25
No	3	18.75
Do you believe that food borne diseases are preventable?		
Yes	13	81.25
No	3	18.75
Do you agree that raw meat can be contaminated through cross contamination with food handlers?		
Strongly agree	8	50
Agree	3	18.75
Strongly disagree	3	18.75
No opinion	2	12.5
Are insects such as cockroaches and flies might transmit food borne pathogens?		
Yes	13	81.25
No	1	6.25
I don’t know	2	12.5
Are you carried out your work with hand glove appropriately during slaughtering?		
Yes	3	18.78
No	13	81.25
Are you wearing gown and a hairnet during slaughtering?		
Yes	12	75
No	4	25
Water source used to wash the carcass in slaughterhouse		
Communal distribution		
Tap water		
Tanker	X	

On the other hands, as the observational checklist (Table 7) showed and also the slaughtering workers agreed that the slaughterhouse is very narrow and sometimes it is not comfort to slaughtering, for example, there is not well organized class/partitioned/ in the slaughterhouse even the floor and wall not appropriate. There are no latrine facilities as the whole in the slaughtering area and sometimes shortage of water which used to washing of carcass and maintaining of the workers personal hygiene was observed. As we and also the slaughtering workers agreed that, the refuse receptacles in the slaughter house were not well established and even if it is in, it did not so far from the slaughtering area. This enhances the carcass to easily expose to flies and other insect vectors that carries and transmit pathogens. At the end of slaughtered, the carcass has been transported to retailers (butchers) of Assosa Town through car. However, the way of transportation of the carcass still were not safe (unsatisfactory) because as we observed in the slaughtering area the carcass could not kept properly in which the carcass were contaminated with slaughtering workers bare hands and shoulders and even the carcass exposed to vectors up to the butcher sites. As a result such practices will enhance the higher microbial (pathogens) load on the carcass. So, the concerning body should give attention as awareness creation among slaughtering workers especially during transportation to cease such types of inappropriate (unsafe) practices.

**Table 7:** Slaughterhouse environment observation in Assosa Town, 2018

**Characteristics**	**Frequency *n*=16**	**Percent**
Is the meat (carcass) covered and kept properly and safe during transportation?		
Yes	11	68.75
No	5	31.25
Is the slaughterhouse making comfort to carry activities with cleaned floor, wall or light?		
Agree	12	75
Disagree	12	75
Are you believed that there is appropriate drainage system for collection of liquid waste?		
Agree	12	75
Disagree	12	75
Are you agree that refuse receptacles far from the slaughterhouse?		
Agree	8	50
Disagree	8	50
Is the carcass in Slaughterhouse easily exposed to harbor vectors such as flies & insects?		
Agree	3	18.75
Disagree	13	81.25
Is there any difficulty to achieve slaughtering in the working area?		
Yes	14	87.5
No	2	12.5
Ways of transportation of the carcass from slaughterhouse to the retail outlets	Car	

## Conclusion and recommendation

### Conclusions

In this investigation, majority of the food samples were within acceptable and some were satisfactory quality range but still it indicates that high microbial contamination of the raw beef meat especially those was sold by butchers. Percentage of rejected level of raw beef meat samples was higher at butchers shop for both AMB (30.8%) and *S. aureus* (40%) than slaughter house AMB (13%) and *S. aureus* (11.43%). Similarly, high count of *E. coli*, mean AMB and total coliforms were obtained from Butchers shop. The presence of coliforms, *E. coli*, *S. aureus* and aerobic mesophilic bacteria indicate a possible post contamination and poor microbial quality of the foods. Prevalence of Salmonella was relatively less recorded 9(25.7%) at slaughterhouse than butchers’ shop 12(34.3%). But, still the result showed that there is a need for hygiene to keep safety of meat and following up the health of the animal in order to reduce contamination of meat and its products by pathogens. Most of the butchers and slaughter man lack adequate training on food hygiene and safety as well as the slaughterhouse in the town are not well comfort and well established. So, adequate training to butchers and slaughter man should be given and more emphasis on the re-establishment of the slaughterhouse is needed by Assosa Town Administration office and other concerning body.

### Recommendations

Based on the present study observations the following points will be recommended:

- ◦ Poor hygiene practiced by butchers may lead to the introduction of pathogenic micro-organisms into the products. Therefore, provision of beef meat of good hygienic quality is desirable from consumer health point of view.
- ◦ There is a need to educate retailers and slaughter workers on proper training on food safety knowledge, sanitation, personal hygiene and handling of the raw beef meat.
- ◦ Food handlers in butchers shop and retail outlets must undergo screening examinations every three or four months or at any time requested.
- ◦ The butchers should not handle money while they are preparing and serving raw beef meat to consumers.
- ◦ The slaughtering workers should wear hairnet and glove before or during slaughtering
- ◦ The raw beef meat during transportation and at time of retail in butchers shop should be covered properly
- ◦ Proper cleaning of hands, utensils, equipment and surfaces of cutting board before handling of beef meat should be practiced.
- ◦ Improve the skill level of slaughtering workers especially who done the evisceration process
- ◦ Those who supply low quality meat to the market should be undertaking some quality measures and awareness creation among them about safety handling practice of beef meat.
- ◦ Proper beef meat inspection, screening and eradication of sick and unhealthy animals should be strictly adhered and its operation should be able to limit or control excess contamination of raw beef meat.
- ◦ Local authorities (Town Abattoir Administration office) should provide the workers in slaughterhouse with appropriate sanitary facilities where they can carry out their activities such as supply of potable water, proper waste receptacles, and latrines around the slaughterhouse.
- ◦ Further researches in the future should be conducted on some anti-bacterial susceptibility test to pathogenic organisms isolated from beef meat samples and more emphasis on the health examination of live animals ready for slaughtering.

### Supplementary Information

**Additional file 1: Appendix.** 

## Data Availability

Data supporting the findings of this study are available within the article and its supplementary information files, from the corresponding author upon reasonable request and from the organization.
