# Supplementary material for: Assessment of bacteriological quality and safety of raw meat at slaughterhouse and butchers’ shop (retail outlets) in Assosa Town, Beneshangul Gumuz Regional State, Western Ethiopia
Source: BMC Microbiol. 2023 Dec 19;23:403. doi: 10.1186/s12866-023-03106-2 (PMC10731869; doi:10.1186/s12866-023-03106-2)
Supplement: Supplementary file 1 — Additional file 1: Appendix. [file 12866_2023_3106_MOESM1_ESM.doc]

**APPENDIX**

**Interview (semi structured) questions to assess knowledge of Butchers’ in Assosa town, 2018**

1. Educational status □ Illiterate □ Elementary □High school □College/University
2. Do you know about food borne disease? Yes □ No □

If yes, what is your source of information …………………………………………

1. Have you taken any training on food hygiene and safety? Yes □ No □
2. Do you work when you have diarrhea? Yes □ No □
3. Do you know reason for food contamination ? Yes □ No □

If yes, please specify ………………………………………………

1. Do infect carrier butcher or meat handlers cause food borne illness ? Yes □ No □
2. Do you know that foods borne disease are preventable? Yes □ No □ If yes, how…………………………………………………………………
3. Do you agree that raw meat can be contaminated through cross contamination with food handlers?

□ strongly agree □Agree □ No opinion □ strongly disagree

1. Food borne pathogens can be seen by eye? Yes □ □ No
2. Are insects such as cockroaches and flies might transmit food borne pathogens? Yes □ No □
3. Apparently healthy food handlers might carry food borne pathogen? Yes □ No □

**Observation checklist to assess handling practices and surrounding environments of Retail outlets (Butchers), Assosa town, 2018**

1. Cleaning status of meat contact surface ,equipment or tables

□ protected well □ unprotected

1. Food handlers (butchers) in retail shops were gowns appropriately? Yes □ □ No
2. Food handlers (butchers) in retail shop were a hairnets or hand gloves? Yes □ □ No
3. Finger nails of the meat handlers?

□ Clean &trimmed □ Not trimmed &un clean

1. The carcass or meat in retails shops is stored and kept properly in refrigerator? Yes □ □ No
2. Ways of transportation of the meat carcass from slaughterhouse to the retail outlets

□ Car □ Man labor □ Cart (Gari) □ If others, specify…………

1. Is the meat covered and kept properly during transportation from slaughterhouse to retail shops? Yes □ □ No
2. If any contact of the carcass with the bare hands of the butchers? Yes □ □ No
3. Are their proper solid waste storage receptacles near the vending site?

□ Not available □ Improperly stored □ Proper receptacle available

1. Is the vending area with cleaned floor, wall and adequate lighting? Yes □ □ No
2. Presence of a drainage system for collection and handling of liquid waste? Yes □ □ No
3. Is the carcass in retail shops (outlets) easily exposed to harbor vectors such as flies? Yes □ □ No
4. Is there any discharging from vendor nose ,eye ,ear or cough during visit

□ Observed □ No observed

1. Vendors handling money when vending the raw meat? Yes □ □ No
2. What looks the general hygiene situation of retail shops through observation?

□ Clean (satisfactory) □ Not clean (Un satisfactory) □ If others, specify……………….
